# The Predictive Value of P‐Wave Dispersion and QTc Dispersion in Preeclampsia

**DOI:** 10.1111/anec.70170

**Published:** 2026-02-27

**Authors:** Liting Zhi, Fei Shen, Lili Mei, Jianling Jin, Shili Jiang, Xingmei Huang, Wei Zhu

**Affiliations:** ^1^ Department of Cardiology The First Affiliated Hospital of Soochow University Suzhou China

**Keywords:** gestational hypertension, preeclampsia, pregnant women, P‐wave dispersion, QTc dispersion

## Abstract

**Objective:**

There is growing evidence that pregnant women with gestational hypertension are more likely to develop cardiovascular disease in the future, including arrhythmias. The objective of this study was to analyze changes in electrocardiogram (ECG) parameters in pregnant women with gestational hypertension and identify independent risk factors for preeclampsia.

**Materials and Methods:**

We collected the ECG data of 185 pregnant women and classified them into three groups: the first group was 68 healthy pregnant women in the third trimester, the second group was 61 pregnant women with gestational hypertension (without preeclampsia), and the third group was 56 pregnant women with preeclampsia. P‐wave amplitude, P‐wave dispersion (PWD), P‐wave area (PWA), ventricular activation time (VAT), T‐wave peak‐to‐end interval (Tp‐e interval), maximum QTc interval (QTc max), and QTc dispersion (QTc‐d) were measured.

**Results:**

Ordered logistic regression revealed significant differences in PWD, QTc max, Tp‐e interval, and QTc‐d values among the three groups. However, only PWD and QTc‐d values were significantly higher in the preeclampsia group than in the non‐preeclampsia group by binary logistic regression. Using a cut‐off of ≥ 35.5 ms for the PWD achieved an accuracy of 74.8% in diagnosing preeclampsia (sensitivity 92.86%, specificity 56.59%). Using a cut‐off of ≥ 43.5 ms for the QTc‐d achieved an accuracy of 91.8% (sensitivity 85.71%, specificity 84.50%).

**Conclusions:**

PWD and QTc‐d were identified as independent ECG risk factors for preeclampsia. Monitoring these parameters may help screen high‐risk pregnant women and improve the management of those with a history of gestational hypertension or preeclampsia.

## Graphical abstract

The diagnostic accuracy for preeclampsia was 74.8% using a PWD cutoff of ≥ 35.5 ms and 91.8% using a QTc‐d cutoff of ≥ 43.5 ms. Monitoring these ECG parameters can help identify pregnant women at increased risk.

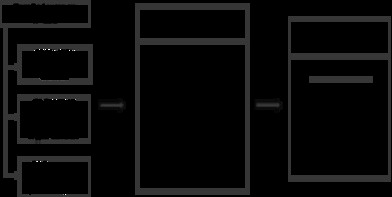

## 1 Introduction

Hypertensive disorders of pregnancy are one of the most significant causes of maternal and fetal death and morbidity, estimated to account for approximately 10% of all pregnancies (Dincgez Cakmak et al. 2019). Gestational hypertension is defined as an increase in blood pressure in the absence of urinary protein after 20 weeks of gestation. Preeclampsia (PE) is a pregnancy‐related multisystem disease that develops after 20 weeks of gestation and is characterized by hypertension with albuminuria or thrombocytopenia, elevated serum creatinine, impaired liver or renal function, pulmonary edema, or cerebral/visual symptoms in the absence of albuminuria, as proposed by the American College of Obstetricians and Gynecologists Task Force on Hypertension in Pregnancy (Hypertension in Pregnancy 2013). PE is the second most common cause of maternal death (10%–15%) (Aksu et al. 2022). In addition, many studies have shown that women with a history of preeclampsia have an increased risk of subsequent cardiovascular and peripheral artery disease. These women with preeclampsia are more likely to develop cardiovascular disease (myocardial infarction, stroke, hypertension) at an earlier age than the general population (Raffaelli et al. 2014). In these studies, pregnant women with hypertension were found to have a 4‐fold increased prevalence of cardiovascular disease and a 2‐fold increased risk of cardiovascular death compared to healthy pregnant women (Ehrenthal et al. 2015; McDonald et al. 2008). In the last few years, several new ECG parameters have been proposed, including the Tp‐e interval, VAT, and P‐wave analysis, and they are associated with increased left ventricular mass, left ventricular diastolic dysfunction, and arrhythmia risk. The QT interval, especially the QTc interval, was considered an indicator of increased cardiovascular risk (Raffaelli et al. 2014). Recent studies have suggested that QT dispersion (QTd) may be a prognostic clinical indicator of mortality in several diseases (Dincgez Cakmak et al. 2019). The objective of this study was to compare the differences in ECG parameters between healthy pregnant women in the third trimester and pregnant women with gestational hypertension and to find independent risk factors for preeclampsia.

## 2 Materials and Methods

### 2.1 Study Population

We collected the clinical, laboratory, and ECG examination data of 185 pregnant women from January 2021 to March 2023 and classified them into three groups: the first group was 68 healthy pregnant women in the third trimester, the second group was 61 pregnant women with gestational hypertension (without preeclampsia), and the third group was 56 pregnant women with preeclampsia. First, the general characteristics were compared among the three study groups. Second, ECG parameters—including P‐wave amplitude, P‐wave dispersion (PWD), P‐wave area (PWA), ventricular activation time (VAT), maximum QTc interval (QTc max), QTc dispersion (QTc‐d), and T‐wave peak‐to‐end interval (Tp‐e interval)—were compared among the three groups using one‐way analysis of variance (ANOVA) and ordered logistic regression. Finally, to identify independent risk factors for preeclampsia, a binary logistic regression analysis was performed. For this analysis, pregnant women were classified into two groups: a preeclampsia group (*n* = 56) and a non‐preeclampsia group (*n* = 129). The non‐preeclampsia group included normotensive pregnant women (*n* = 68) and those with gestational hypertension but without preeclampsia (*n* = 61). The presence or absence of preeclampsia was used as the binary dependent variable.

Gestational hypertension was identified in women with blood pressure exceeding 140/90 mmHg measured at least twice by a mercury sphygmomanometer in the upper arm after 20 weeks of gestation. Preeclampsia was diagnosed after 20 weeks of gestation in women with new‐onset hypertension accompanied by at least one of the following: albuminuria or thrombocytopenia, elevated serum creatinine, impaired liver or renal function, pulmonary edema, or cerebral/visual symptoms without albuminuria. Pregnant women with diabetes, electrolyte imbalance, chronic renal failure, chronic inflammatory disease, chronic lung disease, heart failure or valve disease, thyroid dysfunction, pre‐existing arrhythmia, sleep apnea syndrome, or a history of smoking or drug use during the first trimester were excluded.

### 2.2 ECG Analysis

ECG parameters were derived from a 12‐lead surface electrocardiogram. The paper speed was 25 mm/s, and the amplitude was 10 mm/mV. All ECG parameters were measured manually by two attending physicians, and at least six cardiac cycles were recorded. Interobserver agreement was assessed using the intraclass correlation coefficient (ICC), which showed excellent reliability (ICC for PWD = 0.91; ICC for QTc‐d = 0.89). PWD was defined as the difference between the maximum and minimum P‐wave duration. The Tp‐e interval was defined as the duration from the top of the T wave to the end of the T wave. The QT interval was the duration from the start of the Q wave to the end of the T wave. The corrected QT interval (QTc interval) was then calculated to adjust for heart rate using Bazett's formula: QTc = QT/√RR. QTc‐d was defined as the difference between the QTc max and the minimum QTc interval (QTc min). VAT was defined as the time interval from the start of the Q wave to the top of the R wave. PWA was defined as the P‐wave amplitude multiplied by half of the P‐wave duration in lead II.

### 2.3 Statistical Analysis

Continuous variables were presented as mean ± standard deviation. Differences among the three study groups were compared using one‐way ANOVA. For ECG parameters that showed significant differences in the ANOVA (*p* < 0.05), a sequential multivariate analytical approach was applied. First, ordered logistic regression was used to determine the association between these ECG parameters and the severity of hypertensive disorders. Second, only variables that were significant in the ANOVA were included in a binary logistic regression model, with preeclampsia defined by comparing the preeclampsia group against the non‐preeclampsia group. Finally, to assess the diagnostic utility of the independent risk factors in the binary logistic regression, receiver operating characteristic (ROC) curves were plotted, and the area under the curve (AUC) was calculated for each. All statistical analyses were performed using IBM SPSS Statistics (version 31.0). ROC curves were plotted with GraphPad Prism (version 10.0). A *p*‐value of < 0.05 was considered statistically significant.

### 2.4 Ethics Statement

This study was approved by the Medical Ethics Committee of the First Affiliated Hospital of Soochow University with a waiver of informed consent (Study code 2024–325). All methods were performed in accordance with the relevant guidelines and regulations. All patients gave their oral or written informed consent.

## 3 Results

**FIGURE 1:**
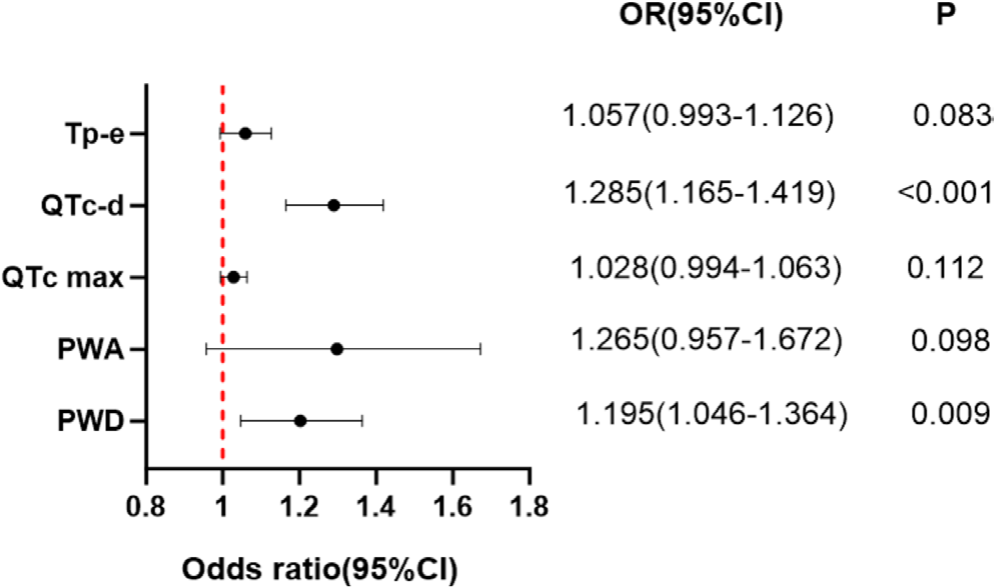
Binary logistic regression between the non‐preeclampsia group and the preeclampsia group.

**FIGURE 2:**
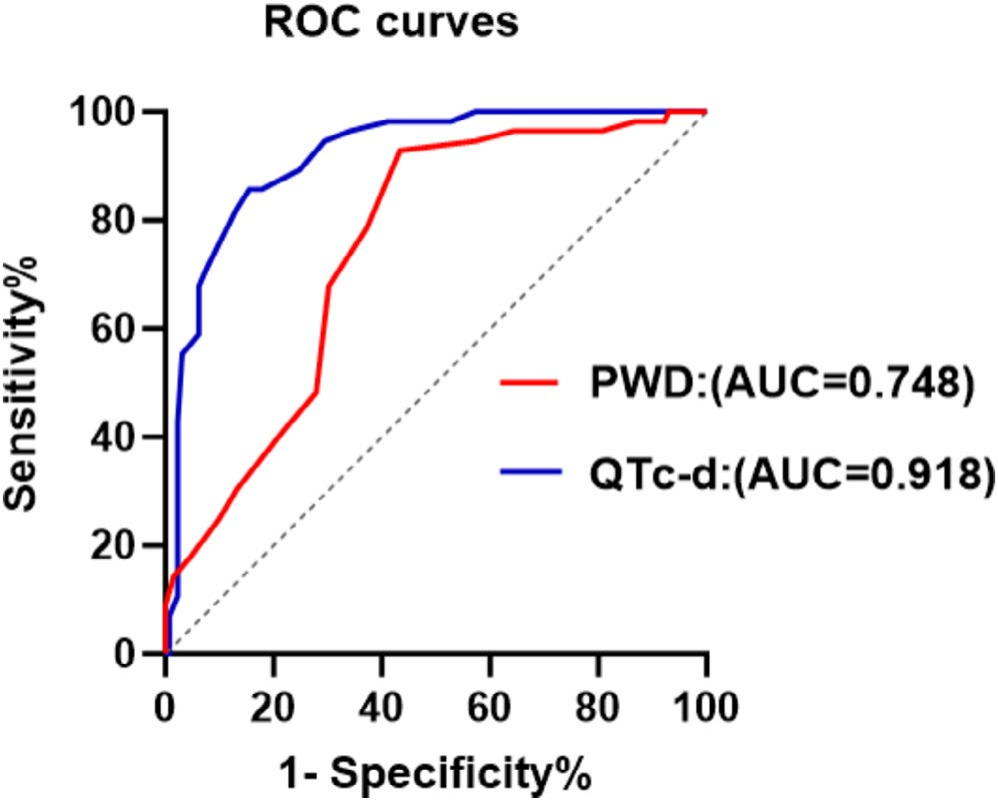
ROC curves of PWD and QTc‐d to predict the presence of preeclampsia.

General characteristics of the three groups are presented in Table 1. There were no significant differences in maternal age, hemoglobin, platelets, total cholesterol, triglyceride, and blood glucose among the three groups. Body Mass Index (BMI) was higher in the gestational hypertension group than in the control group, but there was no significant difference between the control group and the preeclampsia group. Systolic blood pressure and diastolic blood pressure were significantly higher in the gestational hypertension group and the preeclampsia group than in the control group, with no significant difference observed between the two hypertensive groups. ECG findings of the three groups are reported in Table 2. There were no significant differences in P‐wave amplitude and VAT, while significant differences among the groups were observed for PWD, PWA, QTc max, QTc‐d, and Tp‐e interval. PWD, PWA, QTc‐d, QTc max, and Tp‐e interval were analyzed by ordered logistic regression. The results are presented in Table 3. There were no significant differences among the three groups with regard to PWA. Conversely, the association of PWD, QTc‐d, QTc max, and Tp‐e interval showed statistically significant differences among the three groups. Subsequently, PWD, PWA, QTc‐max, QTc‐d, and Tp‐e were included in the binary logistic regression analysis. The results of binary logistic regression between the preeclampsia group and the non‐preeclampsia group are presented in Figure 1. PWD and QTc‐d were significantly higher in the preeclampsia group than in the non‐preeclampsia group. ROC curves for PWD and QTc‐d were generated using SPSS and plotted with GraphPad Prism. The ROC curves are shown in Figure 2. An AUC of 0.748 (range, 0.677–0.820) was observed for PWD and 0.918 (range, 0.877–0.958) for QTc‐d in diagnosing preeclampsia. The optimal diagnostic cutoff was ≥ 35.5 ms for PWD, with an accuracy of 74.8%, and ≥ 43.5 ms for QTc‐d, with an accuracy of 91.8%. The sensitivity, specificity, positive predictive value (PPV), and negative predictive value (NPV) of PWD and QTc‐d for predicting preeclampsia are shown in Table 4.

**TABLE 1:** General characteristics of the three study groups.

Parameters	Control group (*n* = 68)	Gestational hypertension (*n* = 61)	Preeclampsia (*n* = 56)	*p*		
				1 VS 2	1 VS 3	2 VS 3
Maternal age (years)	30.48 ± 5.84	30.13 ± 4.76	31.23 ± 5.52	1.000	1.000	0.817
BMI (kg/m^2^)	27.28 ± 5.55	29.71 ± 6.35	28.61 ± 3.85	0.034	0.520	0.809
Hemoglobin (g/L)	117.85 ± 11.73	118.93 ± 10.29	116.37 ± 18.35	0.930	0.943	0.758
Platelet (×10^9^/L)	188.35 ± 45.57	196.79 ± 55.99	172.88 ± 57.33	1.000	0.351	0.058
Total cholesterol (mmol/L)	6.21 ± 1.16	5.95 ± 1.19	6.17 ± 1.53	0.754	1.000	1.000
Triglyceride (mmol/L)	3.29 ± 1.67	3.34 ± 0.96	3.17 ± 1.28	0.996	0.958	0.813
Fasting glucose (mmol/L)	4.21 ± 0.67	4.25 ± 0.74	4.39 ± 1.22	0.981	0.707	0.860
Systolic BP (mmHg)	115.84 ± 11.33	140.75 ± 6.97	143.96 ± 8.69	< 0.001	< 0.001	0.089
Diastolic BP (mmHg)	75.52 ± 8.13	90.57 ± 7.56	91.07 ± 14.10	< 0.001	< 0.001	1.000

Abbreviation: BMI, Body Mass Index.

**TABLE 2:** ECG findings of the three study groups.

Parameters	Control group (*n* = 68)	Gestational hypertension (*n* = 61)	Preeclampsia (*n* = 56)	*p*		
				1 VS 2	1 VS 3	2 VS 3
P‐wave amplitude	0.12 ± 0.03	0.11 ± 0.04	0.11 ± 0.04	1.000	0.306	1.000
PWD	34.29 ± 4.52	36.39 ± 3.99	39.16 ± 3.69	0.013	< 0.001	0.001
PWA	4.77 ± 1.49	4.86 ± 1.69	5.75 ± 1.94	1.000	0.005	0.015
VAT	38.10 ± 2.74	38.15 ± 2.06	38.55 ± 2.50	1.000	0.936	1.000
QTc max	419.13 ± 13.27	434.79 ± 15.74	435.34 ± 12.95	< 0.001	< 0.001	1.000
QTc‐d	33.18 ± 4.06	41.75 ± 5.60	48.13 ± 4.54	< 0.001	< 0.001	< 0.001
Tp‐e interval	79.26 ± 8.66	89.52 ± 7.64	94.59 ± 6.67	< 0.001	< 0.001	0.002

Abbreviations: PWA, P‐wave area; PWD, P‐wave dispersion; QTc‐d, QTc dispersion; QTc max, Maximum QTc interval; Tp‐e interval, T‐wave peak‐to‐end interval; VAT, Ventricular activation time.

**TABLE 3:** Results of ordered logistic regression analysis among the three study groups.

Parameters	*p*	95% Confidence interval (CI)	
		Lower limit
Upper limit
PWD	0.003	0.047	0.240
PWA	0.243	−0.090	0.356
QTc max	< 0.001	0.024	0.075
QTc‐d	< 0.001	0.224	0.395
Tp‐e interval	0.001	0.032	0.131

Abbreviations: PWA, P‐wave area; PWD, P‐wave dispersion; QTc‐d, QTc dispersion; QTc max, Maximum QTc interval; Tp‐e interval, T‐wave peak‐to‐end interval.

**TABLE 4:** Sensitivity, specificity, positive, and negative predictive values of ECG findings for preeclampsia.

Parameters	Sensitivity	Specificity	PPV	NPV
PWD	92.86%	56.59%	48.15%	94.81%
QTc‐d	85.71%	84.50%	70.59%	93.16%

Abbreviations: NPV, Negative predictive value; PPV, Positive predictive value; PWD, P‐wave dispersion; QTc‐d, QTc dispersion.

## 4 Discussion

In the general data, the BMI was higher in the gestational hypertension group than in the control group, with no significant difference between the control and preeclampsia groups. This means that obesity may cause gestational hypertension, but it may not be a promoting factor for preeclampsia. In ordered logistic regression, PWD, QTc max, QTc dispersion, and Tp‐e interval were significantly higher in pregnant women with gestational hypertension and preeclampsia than in the control group, indicating that these ECG parameters are associated with hypertensive disorders of pregnancy. In binary logistic regression, only PWD and QTc‐d were the independent risk factors for preeclampsia.

QTc‐d reflects ventricular repolarization heterogeneity, and elevated QTc‐d is a known risk factor for arrhythmias. It has been suggested that QTc‐d can be used to identify patients at increased risk of ventricular arrhythmia (Kirbas et al. 2016). It also points out that women with preeclampsia had a longer QTc interval and QTd than normal healthy pregnant women, and preeclampsia was the sole determinant of QTd in the study of Ricciarda Raffaelli et al. (Raffaelli et al. 2014). PWD is associated with the non‐uniform, discontinuous distribution of sinus impulses. Prolonged PWD is a marker of impaired electrical and structural function of the left atrium, which may lead to atrial fibrillation. Although PWD has been less studied in pregnant women with preeclampsia, a case–control study including 58 pregnant women with preeclampsia and 30 healthy pregnant women in their third trimester demonstrated that PWD was significantly increased in the preeclamptic group (Kirbas et al. 2014). A study of 26 pregnant women with preeclampsia and 24 age‐matched healthy pregnant women also found that higher PWD was found in the preeclampsia group. Moreover, PWD was significantly positively associated with atrial and intra‐atrial electromechanical delays (İnci et al. 2015).

In our study, pregnant women with gestational hypertension, especially preeclampsia, had a high PWD and QTc‐d. Therefore, our findings suggest that women with gestational hypertension, particularly those with preeclampsia, face a substantially elevated risk of developing atrial or ventricular arrhythmia. We also found that the accuracy of PWD ≥ 35.5 ms in the diagnosis of preeclampsia was 74.8%, and the accuracy of QTc‐d ≥ 43.5 ms in the diagnosis of preeclampsia was 91.8%. The sensitivity and specificity of PWD for predicting preeclampsia were 92.86% and 56.59%. The sensitivity and specificity of QTc‐d for predicting preeclampsia were 85.71% and 84.50%. Pregnant women exhibiting both a PWD ≥ 35.5 ms and a QTc‐d ≥ 43.5 ms have a high probability of developing preeclampsia. Therefore, monitoring PWD and QTc‐d can help us screen out pregnant women at high risk promptly and remind us to pay more attention to the clinical changes and treatment of such pregnant women to avoid the occurrence of preeclampsia. In the future, follow‐up studies on the occurrence of arrhythmias in women with PWD ≥ 35.5 ms and QTc‐d ≥ 43.5 ms could clarify their actual risk.

This study has several limitations that should be acknowledged. First, it was a single‐center study with a modest sample size. The proposed cutoff values (PWD ≥ 35.5 ms and QTc‐d ≥ 43.5 ms) require validation in larger, multi‐center cohorts. Second, as a cross‐sectional study design, it establishes an association but does not prove causality or the predictive efficacy of these ECG parameters for preeclampsia. Prospective studies are needed to confirm their predictive value. Finally, although ECG parameters were measured manually by experienced physicians, the possibility of inter‐observer variability cannot be entirely ruled out. Future studies could benefit from automated or semi‐automated measurement methods to improve reproducibility.

## Author Contributions

All authors contributed significantly to this study. Wei Zhu designed the study. Xingmei Huang and Shili Jiang collected the data. Fei Shen and Lili Mei analyzed the data. Jianling Jin made the Tables and Figures. Liting Zhi and Fei Shen wrote the paper. All authors reviewed and approved the manuscript. Liting Zhi and Fei Shen contributed equally to this work.

## Funding

The authors have nothing to report.

## Conflicts of Interest

The authors declare no conflicts of interest.

## Data Availability

The raw data supporting the conclusion of this article will be made available by the corresponding author.
